# A network meta-analysis evaluating valgization high tibial osteotomy cutting guides: improving surgical precision through navigation and PSI

**DOI:** 10.1186/s43019-025-00278-1

**Published:** 2025-06-18

**Authors:** Fanny Delaigue, Hassan Wardani, Jules Descamps, Matthieu Ollivier, Rémy Nizard, Pierre-Alban Bouché

**Affiliations:** 1 Orthopedic and Traumatology Surgery Department, Unité de Chirurgie Orthopédique et Traumatologique, Lariboisière Hospital, Aile jaune 2eétage, 2 Rue Ambroise Paré, 75010 Paris, France; 2 Institute for Locomotion, Aix-Marseille University, Marseille, France

**Keywords:** Osteotomy, Genu varum, Network meta-analysis, Tibial valgus osteotomies, Cutting guides

## Abstract

**Background:**

A total of three techniques are used to guide tibial cuts in high tibial osteotomy (HTO): the conventional method, navigation systems, and patient-specific instrumentation (PSI). This network meta-analysis sought to assess whether any of these methods achieve better radiological outcomes, greater functional gains, or a reduced rate of complications.

**Design:**

We included all controlled and noncontrolled trials comparing at least two of the surgical techniques. Primary outcomes were rates of medial proximal tibial angle (MPTA) and posterior tibial slope (PTS) outliers. Secondary outcomes included the rate of hip-knee-ankle (HKA) angle outliers, joint range of motion, postoperative clinical scores, and complication rates.

**Results:**

The analysis included 24 studies with 1817 patients and 1951 operated knees. PSI did not reduce the rate of MPTA outliers compared with conventional techniques (95% credible intervals, CI [0.09–56.84]) or navigation (95% CI [0.03–25.62]), and navigation did not reduce the rate compared with conventional methods (95% CI [0.84–9.17]). Navigation reduced the rate of PTS outliers compared with conventional techniques (95% CI [1.93–1.56.10^4^]). No study investigating PTS outliers with PSI was identified or included. Both navigation and PSI reduced the rate of HKA angle outliers (95% CI [1.33–3.16] and [1.15–42.61], respectively). Aside from the rate of HKA angle outliers and the Lysholm score between 1 and 2 years postoperatively, no differences were observed for other outcomes.

**Conclusions:**

Navigation and PSI allow for more precise achievement of the PTS and HKA angle values set by the surgeons but do not affect long-term knee function or complication rates. However, the cost and limited availability of these techniques should be considered, especially in the absence of additional functional benefits.

**Supplementary Information:**

The online version contains supplementary material available at 10.1186/s43019-025-00278-1.

## Introduction

The success of high tibial osteotomy (HTO), whether open- or closed-wedge, depends on proper patient selection and surgical precision, particularly in achieving the desired frontal femorotibial alignment [1]. Traditionally, the mechanical axis of the lower limb has been aligned to pass through the Fujisawa point, located at 62.5% of the tibial plateau, aiming for a slight overcorrection with 3–5° of valgus postoperatively [2, 3].

The oldest and most widely used method to achieve HTO is the conventional freehand technique, where the osteotomy is opened or closed on the basis of preoperative planning, typically following the Miniaci or Dugdale methods [4–6]. Another approach, known as the “1 mm equals 1 degree of correction” rule, can also be used, as well as the Hernigou table, based on trigonometric principles [7]. This conventional technique can be supplemented by intraoperative methods, such as the cable method or the use of a radiopaque grid, to verify the anatomical alignment of the lower limb [5, 8–12]. However, this conventional method only allows control of the deformity in the frontal plane and is therefore at risk of altering the posterior tibial slope (PTS). Additionally, its accuracy varies depending on the surgeon’s experience, with a risk of undercorrection, leading to persistent pain and risk of recurrence of the deformity [1].

In this context, several techniques have been developed to improve the accuracy of HTO to achieve the correction goals set by the surgeon. A more recent approach involves the use of navigation systems, which provide real-time intraoperative control of three-dimensional (3D) corrections through a sensor system attached to the femur and tibia. Additionally, patient-specific instrumentation (PSI) has been developed, utilizing preoperative data from computed tomography (CT) or magnetic resonance imaging (MRI) scans to design customized cutting guides. These guides help achieve the precise position and opening required for osteotomy based on the desired outcomes.

Although advanced techniques such as navigation and PSI have the potential to enhance surgical precision, particularly in the sagittal plane, most meta-analyses have compared only two cutting guides at a time, without providing clear evidence of their benefits. While some studies have reported greater precision with these techniques [8, 10, 13], this has not been consistently confirmed, particularly for PSI [13–16]. Consequently, no consensus has been established regarding the relative accuracy of each cutting guide or which should be preferred. Given this uncertainty, we conducted the first literature-based network meta-analysis to compare these three surgical techniques—conventional, navigation, and PSI—focusing on radiological outcomes, surgical performance (complications and operating time), and postoperative quality of life assessed by functional scores. Our primary hypothesis is that navigation and PSI improve surgical precision compared with the freehand technique.

## Methodology

This systematic literature review and network meta-analysis was registered in International Prospective Register of Systematic Reviews (PROSPERO) following Preferred Reporting Items for Systematic Reviews and Meta-analyses (PRISMA) guidelines.

### Research strategy

We searched for all randomized and nonrandomized clinical trials in electronic databases (Medline, CENTRAL and Embase) starting at the inception date up to 1 September 2024, using dedicated search equations adapted to each database. We manually searched major international orthopedic journals (*The Journal of Bone and Joint Surgery*, *Knee Surgery Sports Traumatology Arthroscopy*, and *The Journal of Arthroplasty*) and conference proceedings of orthopedic meetings from 1 January 2012 to 1 September 2024. We also searched ClinicalTrials.gov and the World Health Organization’s International Clinical Trials Registry Platform (ICTRP). Finally, we systematically screened reference lists of systematic reviews and meta-analyses for any additional references.

### Study eligibility and selection process

All randomized and nonrandomized clinical trials that compared at least two different cutting guides—conventional technique, navigation, or PSI—and reported results for at least one relevant outcome were included. There were no restrictions on evidence level, language, or publication date. Exclusion criteria included studies focusing on osteoarthritis of compartments other than the medial tibiofemoral compartment and studies using anatomical specimens.

### Primary endpoints

The primary endpoints were the rates of outliers for the medial proximal tibial angle (MPTA) and PTS. Each included study defines outliers as values falling outside a range centered around the target correction value determined by surgeons on the basis of radiographic measurements, with target values generally corresponding to those considered normal for each parameter. Given the variability in MPTA and PTS outlier definitions across studies, we chose to consider only the percentages of values classified as aberrant by each author for each parameter.

### Secondary endpoints

Radiological secondary outcomes included the rate of postoperative hip-knee-ankle (HKA) angle outliers. Functional secondary outcomes included joint range of motion and functional scores. Three scoring systems were used: the Knee Society Score (KSS), Western Ontario and McMaster Universities Arthritis Index (WOMAC), and Lysholm scores, measured at three time points—6 months, between 1 and 2 years, and beyond 2 years postoperatively.

Additional secondary outcomes included intraoperative and postoperative data such as overall complication rate, operative time, pain levels one day after surgery, intra- and post-operative fracture rates, pseudarthrosis rates, infection rates, and the rate of revision surgeries for hardware removal or conversion to total knee replacement.

### Study eligibility assessment

The eligibility of each study was independently assessed by two independently trained reviewers on the basis of the study’s title, abstract, or full text, when necessary. Any disagreements were resolved by a third independent reviewer.

### Selection and general characteristics of the network

**Fig. 1:**
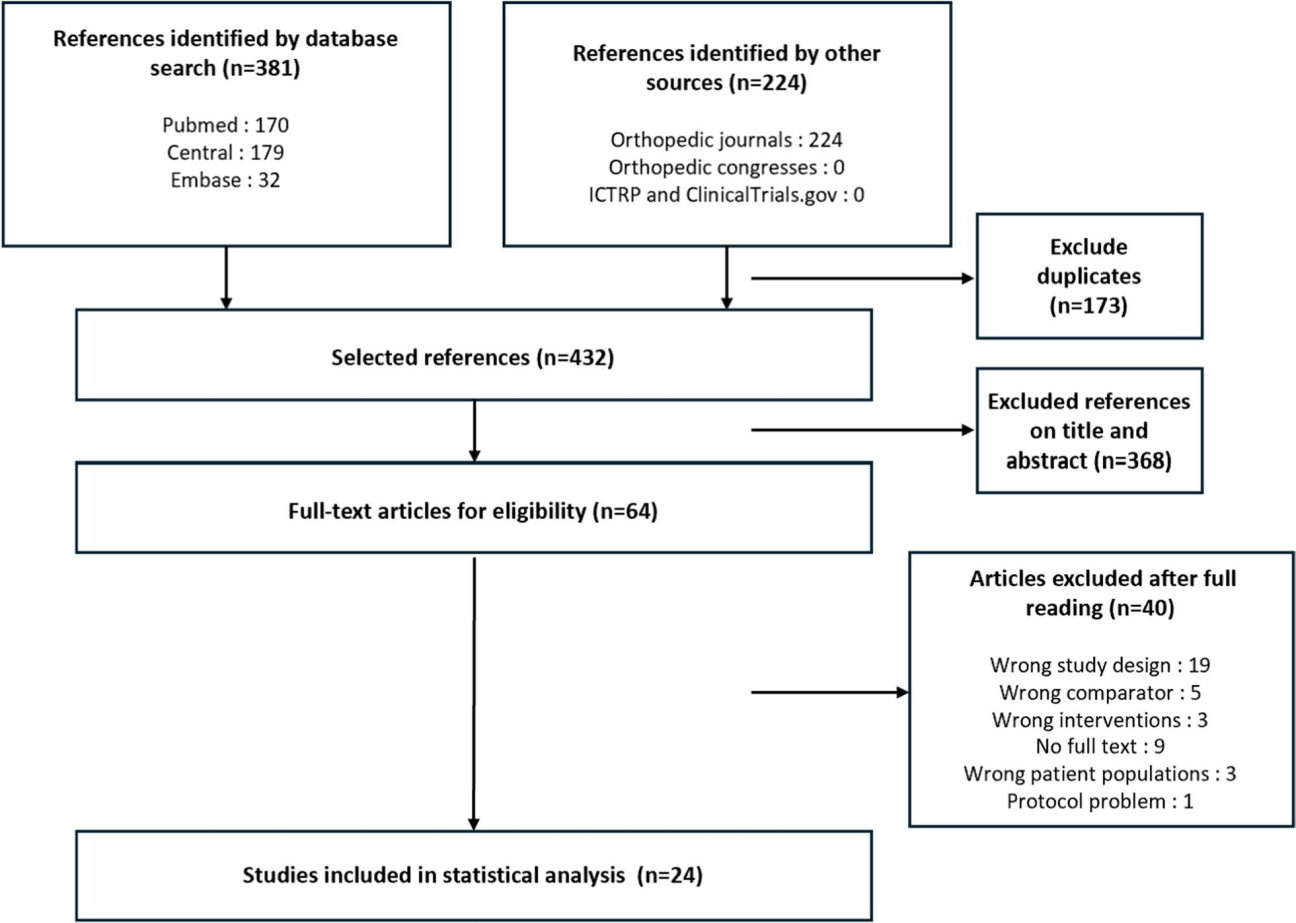
PRISMA flowchart showing the selection process for network meta-analysis

**Fig. 2:**
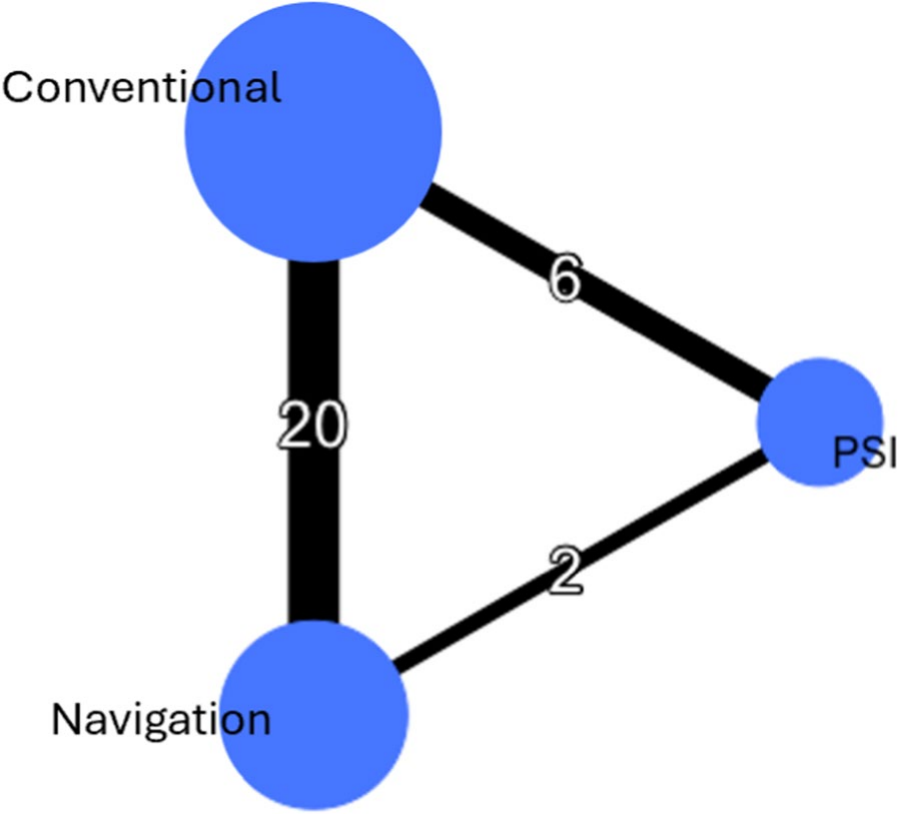
Global meta-analysis network. The different guides are represented by circles (nodes) whose size is proportional to the sample size of each intervention considered. The lines (edges) represent direct comparisons, and their thickness is proportional to the number of articles examining the comparisons

The initial search yielded 605 studies on HTO. Ultimately, 24 randomized and nonrandomized studies were included in the analysis, representing 1817 patients and 1951 operated knees (Fig. 1 for the PRISMA flow chart). The network of studies was closed, with the conventional cutting guide used as the comparator in 100% of the studies (Fig. 2). The primary source of bias was related to confounding factors (Appendix 1). The characteristics of each included study are detailed in Appendix 2.

### Data extraction and risk-of-bias assessment

For each included study, two independent investigators extracted the relevant data and assessed the risk of bias using the Cochrane Risk-of-Bias Tool [17]. An extraction grid was employed after calibration with five randomly selected studies. Any disagreements were resolved by a third independent reviewer. In cases of uncertainty, the authors of the original studies were contacted for clarification.

The extracted data included general study characteristics, patient demographics, details of the cutting guides used, and relevant intervention parameters. For continuous variables, mean values and standard deviations were estimated from the medians, interquartile ranges (first and third quartiles), or minimum and maximum values, following established formulae [18].

### Data analysis

Risk ratios (RRs) were used to compare binary outcomes, while continuous outcomes were analyzed using mean differences (MD) with 95% credible intervals (95% CIs). A traditional meta-analysis was first conducted on the included trials, comparing the different cutting guides using a random-effects model. This allowed the construction of a network for each assessment criterion, which incorporated direct comparisons.

To combine results from both direct and indirect comparisons, we employed a network meta-analysis using Lu and Ades’ hierarchical model with a Bayesian approach [19]. The model was implemented using Markov chain Monte Carlo simulations, with model parameters estimated using Gibbs sampling. Model fit was assessed through residual deviance [20], and network consistency was evaluated using inconsistency factors, as defined by Lu and Ades. Continuous outcomes were analyzed using standardized mean differences, while binary outcomes were assessed using risk differences. All outcomes were reported with 95% CIs.

The treatment effect was ranked for each assessment criterion using Bayesian probabilities. To assess the consistency of the network meta-analysis, the *I*^2^ statistical method was applied. Additionally, local consistency was evaluated using node splitting and loop-based tests [20]. Heterogeneity that could not be attributed to random error was evaluated for each comparison using the *I*^2^ method with its corresponding 95% CI. Random error heterogeneity was assessed using the Cochrane Q test and its 95% CI. Data analysis was performed using R software version 4.3.4 (R Foundation for Statistical Computing, Vienna, Austria). Finally, the validity of the meta-analysis was evaluated using the Grading of Recommendations, Assessment, Development and Evaluation (GRADE) framework [21]. 

## Results

### MPTA outliers

**Fig. 3:**
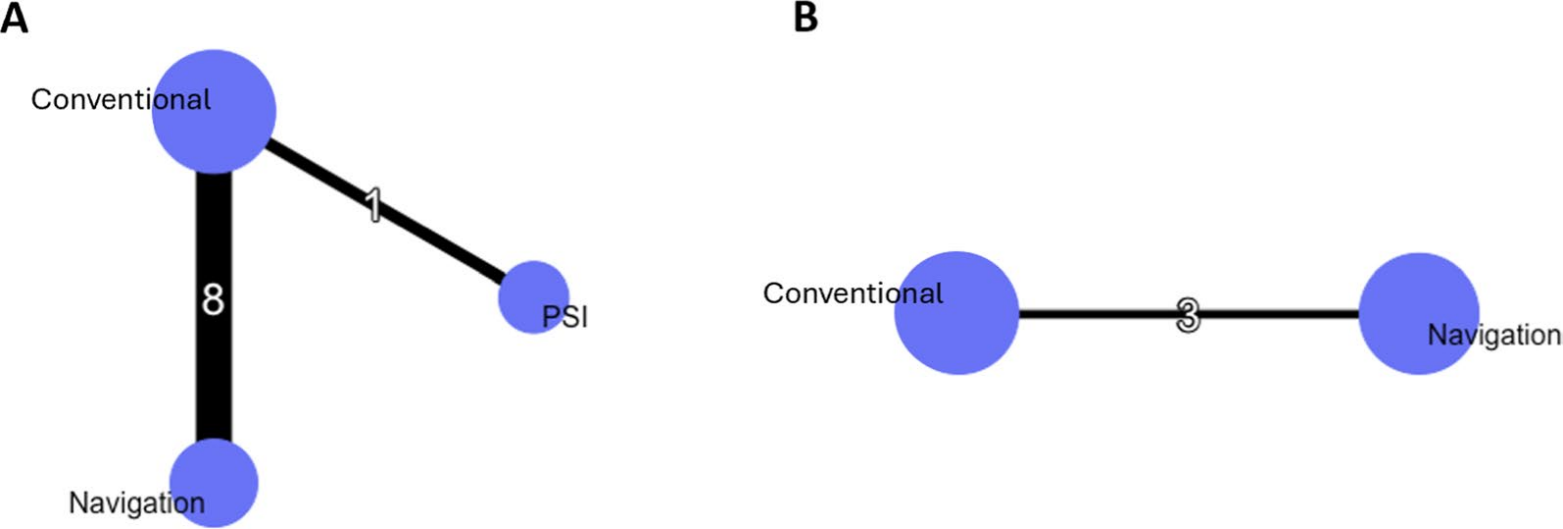
Network plots of key outcomes. (**A**) MPTA outlier rate; (**B**) PTS outlier rate

**Fig. 4:**
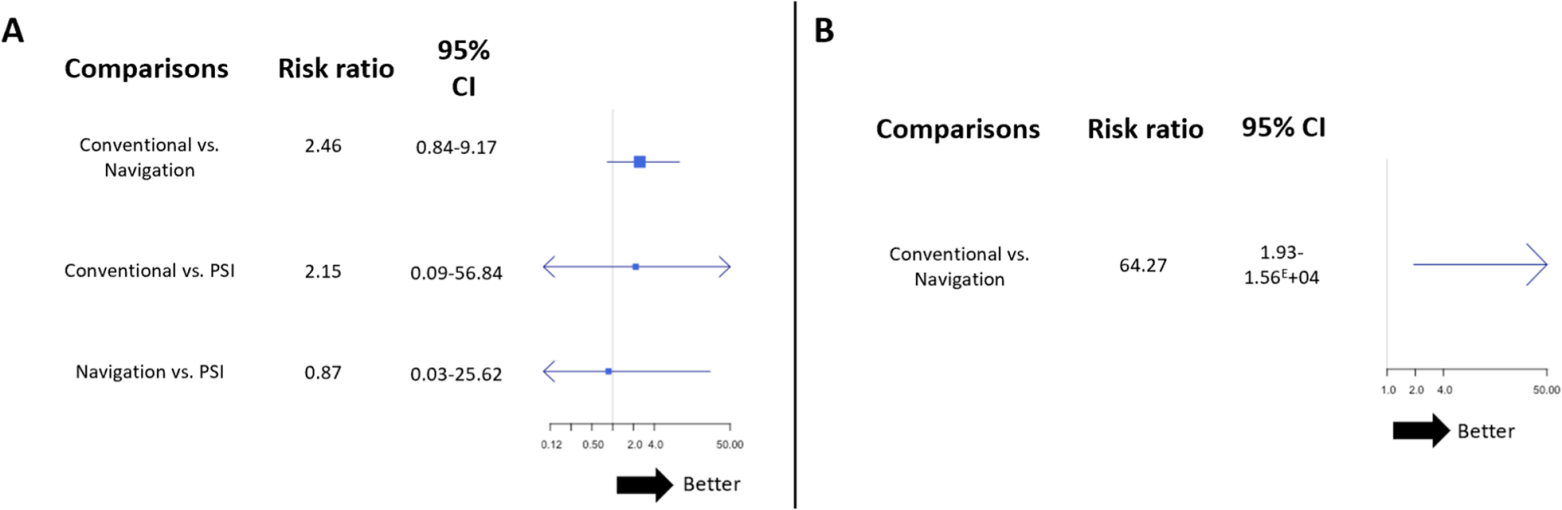
Forest plots of (**A**) MPTA outlier rate and (**B**) PTS outlier rate. Data are presented as risk ratios with 95% credibility intervals. The *I*^2^ are 2% for the MPTA network and 6% for the PTS network

**Fig. 5:**
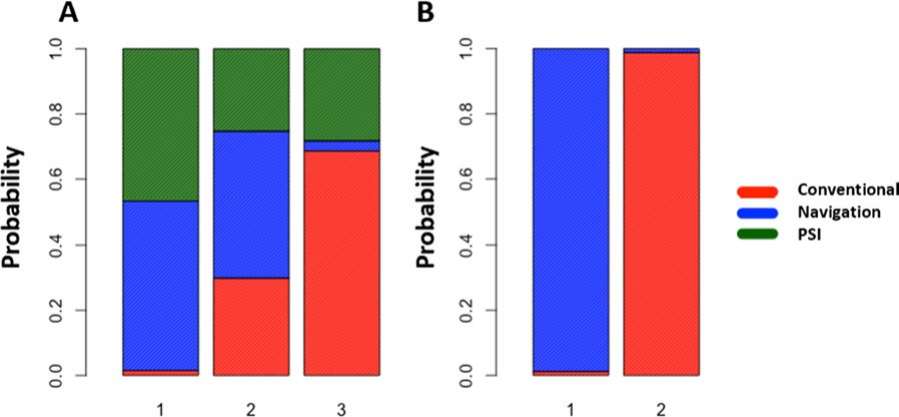
Ranking probabilities of surgical techniques for (**A**) MPTA outlier rate and (**B**) PTS outlier rate. Each guide is represented by a color: green for PSI, blue for navigation, and red for conventional. Ranking is shown on the *x*-axis. The probability of the ranking is shown on the *y*-axis, from 0% to 100%

In total, nine studies reported the rate of postoperative MPTA outliers (Fig. 3A). In total, 100 patients out of 329 (30.4%), 42 out of 267 (15.7%), and 4 out of 36 (11.1%) had aberrant MPTA values after using the conventional method, navigation, and PSI, respectively. The results show that PSI did not reduce the rate of MPTA outliers compared with conventional techniques (RR random effect 2.15; 95% CI 0.09–56.84) or navigation (RR random effect 0.87; 95% CI 0.03–25.62), and navigation similarly did not reduce it compared with conventional methods (RR random effect 2.46; 95% CI 0.84–9.17; Fig. 4A). The *I*^2^ value for the MPTA network was 2.0%, indicating low heterogeneity. PSI had the highest likelihood of achieving the lowest rate of MPTA outliers, followed by navigation and conventional cutting guides (Fig. 5A).

### PTS outliers

In total, three studies reported the rate of postoperative PTS outliers, comparing navigation with conventional methods (Fig. 3B). Moreover, no study examining PTS outliers with PSI was identified or included. In total, 63 patients out of 127 (49.6%) and 4 out of 112 (3.6%) had aberrant PTS values after using the conventional method and navigation, respectively. The results show that navigation reduced the rate of outliers compared with conventional cutting guides (RR random effect 64.27; 95% CI 1.93–1.56.10^4^; Fig. 4B). The *I*^2^ value for the PTS network was 6.0%, indicating moderate heterogeneity. Navigation had the highest probability of reducing the PTS outliers rate compared with conventional techniques (Fig. 5B).

### Secondary endpoints

**Fig. 6:**
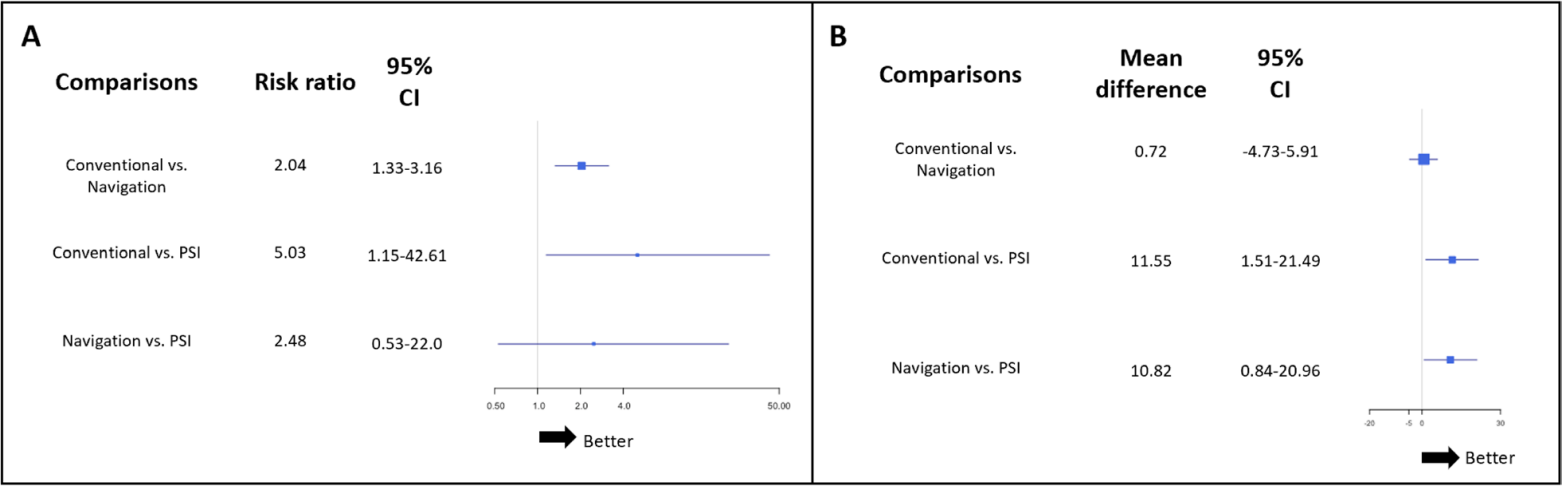
Forest plots of (**A**) HKA angle outlier rate and (**B**) Lysholm score between 1 and 2 years after surgery. Data are presented as risk ratios with 95% credibility intervals. The *I*^2^ was 0% for both HKA angle and Lysholm score networks

For HKA angle outliers’ rate, the findings show that both navigation (RR random effect 2.04; 95% CI 1.33–3.16) and PSI (RR random effect 5.03; 95% CI 1.15–42.61) reduced the rate of HKA angle outliers compared with conventional techniques. However, no significant difference in the outlier rate was observed between PSI and navigation (RR random effect 2.48; 95% CI 0.53–22.0; Fig. 6A).

Regarding clinical outcomes, no significant differences were observed for joint range of motion or functional scores between the methods, except for the Lysholm score between 1 and 2 years postoperatively, which showed improvement with PSI compared with the conventional guide (RR random effect 11.55; 95% CI 1.51: 21.49; Fig. 6B).

No differences were observed in any of the other intra- or post-operative secondary outcomes, including overall complication rates, operating time, pain scores 1 day after surgery, intra- and post-operative fracture rates, pseudarthrosis rates, infection rates, and the rate of revision surgery for hardware removal or conversion to total knee replacement.

### GRADE evaluation

GRADE analyses were performed for each of the primary outcomes. Evidence was low for all comparisons made, i.e., conventional/navigation, conventional/PSI, and navigation/PSI for MPTA angle, and conventional/navigation for the PTS (Appendix 3).

## Discussion

Our results show that navigation significantly reduced the rate of PTS outliers compared with the conventional method. However, the impact of PSI on PTS was not assessed in any of the studies. The rate of MPTA outliers was not reduced by any cutting guide, although PSI had the highest likelihood of reducing MPTA outliers. Similarly, both navigation and PSI reduced the rate of HKA angle outliers in the operated limb compared with the conventional technique. This difference is explained by the fact that the initial value used to calculate the required correction is the HKA measured on goniometry, which accounts for ligamentous laxity and soft tissues that are not considered in the MPTA alone. However, since the correction is only applied to the MPTA, an excessively high lateral distal femoral angle (LDFA) results in an abnormal HKA value. The rankings of cutting guides for radiological outcomes suggest that PSI was the most accurate for achieving optimal alignment in the frontal plane, followed by navigation and the conventional method. While this ranking indicates a trend, it should not be considered a definitive conclusion.

Our network meta-analysis is unique in that none of the existing studies have definitively concluded whether one surgical technique is superior in clinical practice. Indeed, most previous meta-analyses or literature reviews have compared only two cutting guides at a time, and none were conclusive regarding the benefits of navigation or PSI. While several studies reported greater surgical precision with the use of navigation or PSI compared with the conventional technique [8, 10, 13], this was not always confirmed, particularly for PSI [13–16]. One possible explanation for these contradictory results is that HTO was often performed by experienced surgeons, for whom the advantages of PSI in terms of surgical precision or time savings were not evident [13, 14]. Pérez-Mañanes et al. reported a reduction in surgical time and ionizing radiation exposure when PSI was used, without a significant effect on precision in the frontal plane [15]. Our meta-analysis demonstrates an improvement in surgical precision in both the frontal and sagittal planes for navigation, and in the frontal plane for PSI, offering a practical advantage for less experienced operators. Indeed, since the learning curve is shorter for PSI [22–24], they allow for better correction even when the surgeon has limited experience [25]. Moreover, as highlighted by Sys et al. and Leluc et al. [26, 27], PSI may be particularly beneficial for complex osteotomies, such as multiplanar or rotational procedures, where preoperative planning based solely on two dimensions radiographs may be insufficient. Nevertheless, mastering the fundamentals of the freehand technique remains essential to compensate for potential intraoperative failures of navigation or PSI. As we did not identify any postoperative clinical differences regarding the cutting guide used, it remains essential to consider that each surgeon should select the technique they know and master best, while keeping in mind that the freehand method remains the reference standard.

Historically, the standard approach has been to align the postoperative mechanical axis of the lower extremity through the Fujisawa point at 62.5% of the tibial plateau [2, 3]. However, recent studies suggest that better clinical outcomes may be achieved when the axis passes through 50–55% of the tibial plateau [28, 29] or is adjusted on the basis of the degree of preoperative osteoarthritis [30]. In this context, we chose not to report outliers for radiological criteria, as these varied across the included studies, which could enhance the external validity of our findings. We also opted for a Bayesian model, which allowed both direct and indirect comparisons of the different interventions. This approach improved our inferences regarding the relative effectiveness of the cutting guides and enabled us to rank the guides on the basis of treatment probabilities [19].

Apart from the Lysholm score between 1 and 2 years postoperatively, where PSIs outperformed conventional methods, no significant improvements in other clinical scores or joint range of motion were observed on the basis of the cutting guide used. This may be due to the inconsistent use of clinical scoring systems and varying timing of assessments between studies, leading to a lack of consistency in the data. Future studies would benefit from standardized outcome measures, as recommended by the Core Outcome Measures in Effectiveness Trials (COMET) group [31]. Another possible explanation for the lack of clinical improvement could be the heterogeneity of functional measures, which are subjectively reported in clinical scores. The included trials spanned nine different countries across five continents, resulting in varying patient environments [32, 33]. Although this increases the external validity of our study, it reduces statistical power. Additionally, there is no clear threshold for determining when a pre- to post-operative difference in functional outcomes represents a clinically meaningful improvement in a patient’s daily life. To address this, new statistical methods such as minimal clinically important differences (MCIDs) are being developed in clinical research [34, 35].

## Limitations

The study had several limitations. First, both randomized and nonrandomized trials were included to address the limited number of available studies, which may have compromised the homogeneity and coherence of the network meta-analysis. However, we accounted for heterogeneity between studies in terms of population and intervention differences by employing random-effects models, thus avoiding overly precise results with excessively narrow credible intervals, which could lead to false conclusions about treatment differences [36].

Another limitation was the inconsistency in the number of nodes and comparisons within the networks, largely due to the limited number of studies reporting functional scores (only 10 studies, or 41.7% of all studies) and radiological outcomes (17 studies, or 70.8%). The lack of consensus on which functional scores to use following knee surgery and the variability in the target correction angle of 3° valgus after HTO contributed to this heterogeneity [37]. Additionally, only a small proportion of studies provided outlier rates for key radiological endpoints, such as MPTA (30.5%) and PTS (12.5%), and attempts to gather missing data from authors were unsuccessful.

Moreover, means and standard deviations for continuous variables were sometimes missing, requiring estimation using formulae based on the assumption of a Gaussian distribution [18]. The lack of uniformity in measuring functional scores and varying patient follow-up periods may have also influenced the comparison of outcomes and complication rates. Although only one significant difference was found among these criteria, the variations in follow-up timing likely had a minimal overall impact on clinical outcomes.

The limited follow-up duration across studies is another notable limitation, potentially explaining the absence of significant differences in functional outcomes and revision rates between surgical techniques. Hernigou et al. highlighted the emergence of recurrent pain and varying complication rates approximately 7 years post-HTO [38], underscoring the need for long-term research to evaluate the benefits of new cutting guides on knee function and revision rates.

Potential publication bias was also a concern, as no relevant gray literature or oral presentations were included; however, the risk was likely reduced through thorough searches of ClinicalTrials.gov and ICTRP for unpublished trials. Cutting guide rankings should be interpreted as trends rather than definitive results owing to their probabilistic nature, although they do provide useful indications for guide selection. Lastly, economic considerations and equipment availability were not assessed even though it can influence the choice of the cutting guide, and our findings specifically apply to frontal plane monoplanar HTO, limiting generalizability to bi- or tri-planar HTO procedures.

## Conclusions

Navigation and PSI enhanced the precision of valgus tibial osteotomy by reducing PTS outliers and better controlling the HKA angle; however, they did not improve most clinical outcomes or reduce perioperative and postoperative complications. Due to the high costs and limited functional benefits, the routine use of these technologies in clinical practice may be limited.

## Supplementary Information

Supplementary Material 1: Appendix 1: risk of bias of included studies

Supplementary Material 2: Appendix 2: details about included studies. References, general details and surgical details

Supplementary Material 3: Appendix 3: GRADE evaluation for primary outcomes

## Data Availability

The dataset supporting the conclusions of this article is available from the corresponding author on reasonable request.

## Abbreviations

3D: Three-dimensional
95% CI: 95% credible interval
COMET: Core Outcome Measures in Effectiveness Trials
CT: Computed tomography
HKA: Hip-knee-ankle
HTO: High tibial osteotomy
ICTRP: International Clinical Trials Registry Platform
KSS: Knee Society Score
LDFA: Lateral distal femoral angle
MCID: Minimal clinically important differences
MD: Mean difference
MPTA: Medial proximal tibial angle
MRI: Magnetic resonance imaging
PRISMA: Preferred Reporting Items for Systematic Reviews and Meta-analyses
PROSPERO: International Prospective Register of Systematic Reviews
PSI: Patient-specific instrumentation
PTS: Posterior tibial slope
RR: Risk ratio
WOMAC: Western Ontario and McMaster Universities Arthritis Index
