# Supplementary figures and images for: A network meta-analysis evaluating valgization high tibial osteotomy cutting guides: improving surgical precision through navigation and PSI
Source: Knee Surg Relat Res. 2025 Jun 18;37:28. doi: 10.1186/s43019-025-00278-1 (PMC12177990; doi:10.1186/s43019-025-00278-1)

*Appendix 1.*


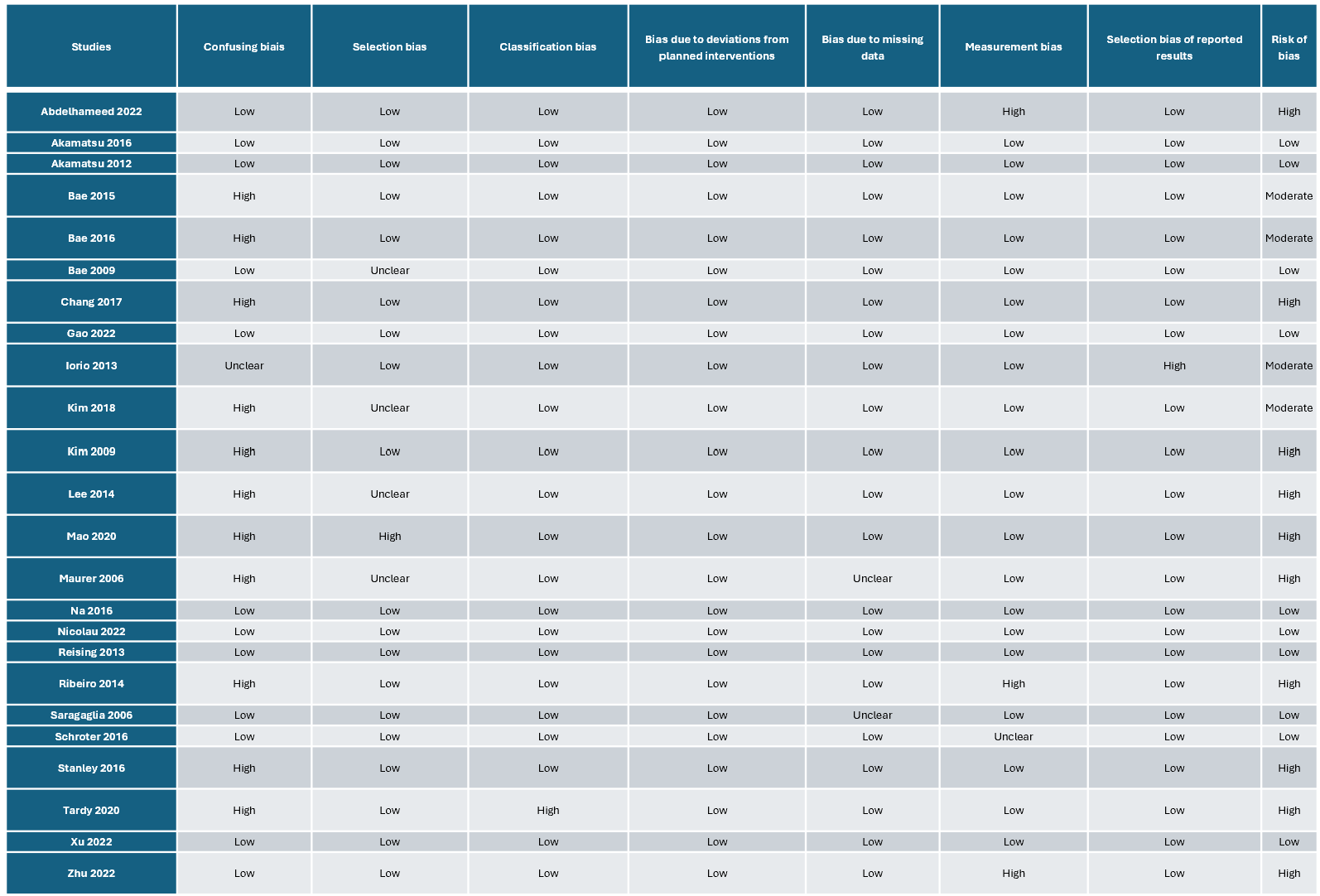

Supplement: Supplementary file 1 — Supplementary Material 1: Appendix 1: risk of bias of included studies [file 43019_2025_278_MOESM1_ESM.docx]

*Appendix 3.*


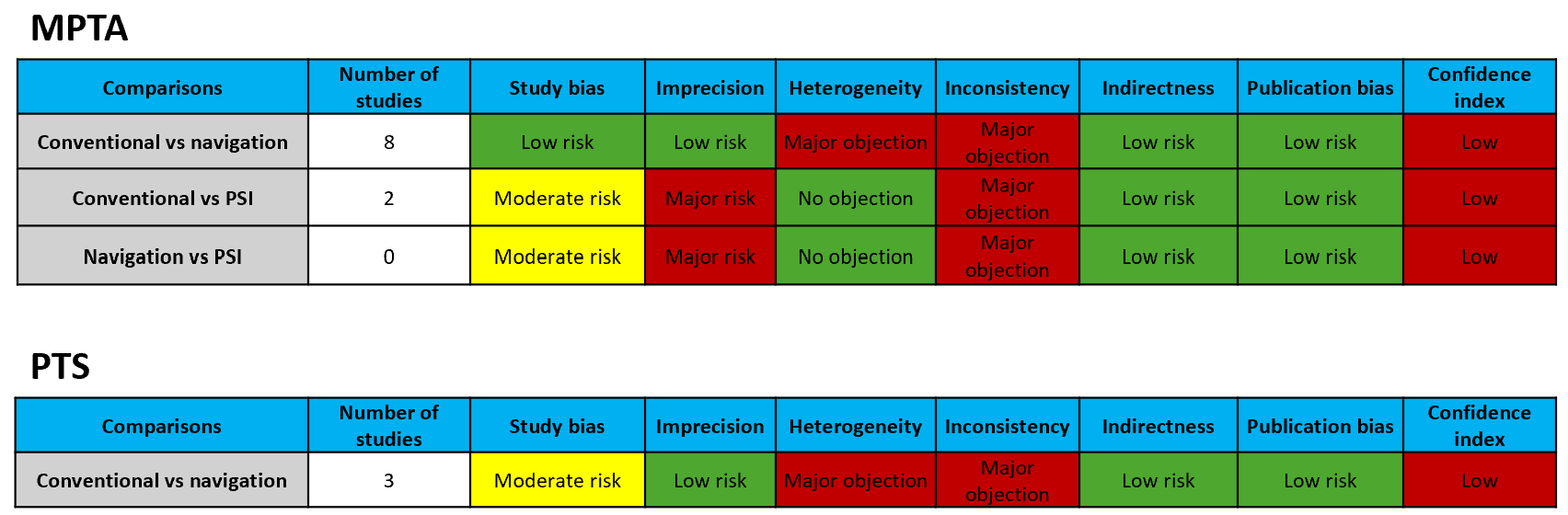

Supplement: Supplementary file 3 — Supplementary Material 3: Appendix 3: GRADE evaluation for primary outcomes [file 43019_2025_278_MOESM3_ESM.docx]
