# Supplementary material for: A network meta-analysis evaluating valgization high tibial osteotomy cutting guides: improving surgical precision through navigation and PSI
Source: Knee Surg Relat Res. 2025 Jun 18;37:28. doi: 10.1186/s43019-025-00278-1 (PMC12177990; doi:10.1186/s43019-025-00278-1)

*Appendix 2.*

1) References

1. Abdelhameed MA, Yang CZ, AlMaeen BN, Jacquet C, Ollivier M. No benefits of knee osteotomy patient’s specific instrumentation in experienced surgeon hands. *Knee Surg Sports Traumatol Arthrosc*. 2023;31(8):3133-3140. doi:[10.1007/s00167-022-07288-6](https://doi.org/10.1007/s00167-022-07288-6)

Non randomized. 84 patients included, 91 HTO : 50 conventional, 41 navigated.

2. Akamatsu Y, Mitsugi N, Mochida Y, et al. Navigated opening wedge high tibial osteotomy improves intraoperative correction angle compared with conventional method. *Knee Surg Sports Traumatol Arthrosc*. 2012;20(3):586-593. doi:[10.1007/s00167-011-1616-8](https://doi.org/10.1007/s00167-011-1616-8)

Non randomized. 62 patients included, 62 HTO : 31 conventional, 31 navigated.

3. Akamatsu Y, Kobayashi H, Kusayama Y, Kumagai K, Saito T. Comparative Study of Opening-Wedge High Tibial Osteotomy With and Without a Combined Computed Tomography-Based and Image-Free Navigation System. *Arthroscopy*. 2016;32(10):2072-2081. doi:[10.1016/j.arthro.2016.02.018](https://doi.org/10.1016/j.arthro.2016.02.018)

Randomized. 48 patients included, 59 HTO : 28 conventional, 31 navigated.

4. Bae DK, Song SJ, Yoon KH. Closed-wedge high tibial osteotomy using computer-assisted surgery compared to the conventional technique. *J Bone Joint Surg Br*. 2009;91(9):1164-1171. doi:[10.1302/0301-620X.91B9.22058](https://doi.org/10.1302/0301-620X.91B9.22058)

Non randomized. 118 patients included, 150 HTO : 75 conventional, 75 navigated.

5. Bae DK, Ko YW, Kim SJ, Baek JH, Song SJ. Computer-assisted navigation decreases the change in the tibial posterior slope angle after closed-wedge high tibial osteotomy. *Knee Surg Sports Traumatol Arthrosc*. 2016;24(11):3433-3440. doi:[10.1007/s00167-016-4032-2](https://doi.org/10.1007/s00167-016-4032-2)

Non randomized. 109 patients included, 150 HTO : 75 conventional, 75 navigated.

6. Bae DK, Song SJ, Kim KI, Hur D, Jeong HY. Mid-term survival analysis of closed wedge high tibial osteotomy: A comparative study of computer-assisted and conventional techniques. *Knee*. 2016;23(2):283-288. doi:[10.1016/j.knee.2015.10.005](https://doi.org/10.1016/j.knee.2015.10.005)

Non randomized. 82 patients included, 100 HTO : 50 conventional, 50 navigated.

7. Chang J, Scallon G, Beckert M, et al. Comparing the accuracy of high tibial osteotomies between computer navigation and conventional methods. *Comput Assist Surg (Abingdon)*. 2017;22(1):1-8. doi:[10.1080/24699322.2016.1271909](https://doi.org/10.1080/24699322.2016.1271909)

Non randomized. 107 patients included, 107 HTO : 66 conventional, 41 navigated.

8. Gao F, Yang X, Wang C, et al. Comparison of Clinical and Radiological Outcomes between Calibratable Patient-Specific Instrumentation and Conventional Operation for Medial Open-Wedge High Tibial Osteotomy: A Randomized Controlled Trial. *Biomed Res Int*. 2022;2022:1378042. doi:[10.1155/2022/1378042](https://doi.org/10.1155/2022/1378042)

Randomized. 37 patients included, 39 HTO : 23 conventional, 16 PSI.

9. Iorio R, Pagnottelli M, Vadalà A, et al. Open-wedge high tibial osteotomy: comparison between manual and computer-assisted techniques. *Knee Surg Sports Traumatol Arthrosc*. 2013;21(1):113-119. doi:[10.1007/s00167-011-1785-5](https://doi.org/10.1007/s00167-011-1785-5)

Non randomized. 24 patients included, 27 HTO : 13 conventional, 14 navigated.

10. Kim HJ, Park J, Shin JY, Park IH, Park KH, Kyung HS. More accurate correction can be obtained using a three-dimensional printed model in open-wedge high tibial osteotomy. *Knee Surg Sports Traumatol Arthrosc*. 2018;26(11):3452-3458. doi:[10.1007/s00167-018-4927-1](https://doi.org/10.1007/s00167-018-4927-1)

Non randomized. 40 patients included, 40 HTO : 20 conventional, 20 PSI.

11. Kim SJ, Koh YG, Chun YM, Kim YC, Park YS, Sung CH. Medial opening wedge high-tibial osteotomy using a kinematic navigation system versus a conventional method: a 1-year retrospective, comparative study. *Knee Surg Sports Traumatol Arthrosc*. 2009;17(2):128-134. doi:[10.1007/s00167-008-0630-y](https://doi.org/10.1007/s00167-008-0630-y)

Non randomized. 85 patients included, 90 HTO : 43 conventional, 47 navigated.

12. Lee DH, Han SB, Oh KJ, et al. The weight-bearing scanogram technique provides better coronal limb alignment than the navigation technique in open high tibial osteotomy. *Knee*. 2014;21(2):451-455. doi:[10.1016/j.knee.2012.09.003](https://doi.org/10.1016/j.knee.2012.09.003)

Non randomized. 78 patients included, 84 HTO : 43 conventional, 41 navigated.

13. Mao Y, Xiong Y, Li Q, et al. 3D-Printed Patient-Specific Instrumentation Technique Vs. Conventional Technique in Medial Open Wedge High Tibial Osteotomy: A Prospective Comparative Study. *Biomed Res Int*. 2020;2020:1923172. doi:[10.1155/2020/1923172](https://doi.org/10.1155/2020/1923172)

Non randomized. 37 patients included, 37 HTO : 19 conventional, 18 PSI.

14. Maurer F, Wassmer G. High tibial osteotomy: does navigation improve results? *Orthopedics*. 2006;29(10 Suppl):S130-132.

Non randomized. 67 patients included, 67 HTO : 23 conventional, 44 navigated.

15. Na YG, Eom SH, Kim SJ, Chang MJ, Kim TK. The use of navigation in medial opening wedge high tibial osteotomy can improve tibial slope maintenance and reduce radiation exposure. *Int Orthop*. 2016;40(3):499-507. doi:[10.1007/s00264-015-2880-x](https://doi.org/10.1007/s00264-015-2880-x)

Non randomized. 60 patients included, 60 HTO : 20 conventional, 40 navigated.

16. Nicolau X, Jenny JY, Bonnomet F, Ollivier M, Favreau H, Ehlinger M. Accuracy of the correction achieved after a valgus high tibial osteotomy: Comparison of the Hernigou table and navigation. *Orthop Traumatol Surg Res*. 2022;108(3):103241. doi:[10.1016/j.otsr.2022.103241](https://doi.org/10.1016/j.otsr.2022.103241)

Non randomized. 40 patients included, 43 HTO : 21 conventional, 22 navigated.

17. Reising K, Strohm PC, Hauschild O, et al. Computer-assisted navigation for the intraoperative assessment of lower limb alignment in high tibial osteotomy can avoid outliers compared with the conventional technique. *Knee Surg Sports Traumatol Arthrosc*. 2013;21(1):181-188. doi:[10.1007/s00167-012-2088-1](https://doi.org/10.1007/s00167-012-2088-1)

Non randomized. 80 patients included, 80 HTO : 40 conventional, 40 navigated.

18. Ribeiro CH, Severino NR, Moraes de Barros Fucs PM. Opening wedge high tibial osteotomy: navigation system compared to the conventional technique in a controlled clinical study. *Int Orthop*. 2014;38(8):1627-1631. doi:[10.1007/s00264-014-2341-y](https://doi.org/10.1007/s00264-014-2341-y)

Non randomized. 38 patients included, 38 HTO : 20 conventional, 18 navigated.

19. Saragaglia D, Mercier N, Colle PE. Computer-assisted osteotomies for genu varum deformity: which osteotomy for which varus? *Int Orthop*. 2010;34(2):185-190. doi:[10.1007/s00264-009-0757-6](https://doi.org/10.1007/s00264-009-0757-6)

Non randomized. 56 patients included, 56 HTO : 28 conventional, 28 navigated.

20. Schröter S, Ihle C, Elson DW, Döbele S, Stöckle U, Ateschrang A. Surgical accuracy in high tibial osteotomy: coronal equivalence of computer navigation and gap measurement. *Knee Surg Sports Traumatol Arthrosc*. 2016;24(11):3410-3417. doi:[10.1007/s00167-016-3983-7](https://doi.org/10.1007/s00167-016-3983-7)

Randomized. 113 patients included, 113 HTO : 57 conventional, 56 navigated.

21. Stanley JC, Robinson KG, Devitt BM, et al. Computer assisted alignment of opening wedge high tibial osteotomy provides limited improvement of radiographic outcomes compared to fluoroscopic alignment. *Knee*. 2016;23(2):289-294. doi:[10.1016/j.knee.2015.12.006](https://doi.org/10.1016/j.knee.2015.12.006)

Non randomized. 111 patients included, 117 HTO : 65 conventional, 52 navigated.

22. Tardy N, Steltzlen C, Bouguennec N, et al. Is patient-specific instrumentation more precise than conventional techniques and navigation in achieving planned correction in high tibial osteotomy? *Orthop Traumatol Surg Res*. 2020;106(8S):S231-S236. doi:[10.1016/j.otsr.2020.08.009](https://doi.org/10.1016/j.otsr.2020.08.009)

Non randomized. 126 patients included, 126 HTO : 61conventional, 26 navigated, 39 PSI.

23. Xu Z, Tian G, Zhang R, Wu Z, Liu C, Ye C. Clinical efficacy and feasibility of laser correction technology with an ordinary laser pen and surgical instrument box in open-wedge high tibial osteotomy. *BMC Musculoskelet Disord*. 2022;23(1):1019. doi:[10.1186/s12891-022-05993-4](https://doi.org/10.1186/s12891-022-05993-4)

Non randomized. 71 patients included, 71 HTO : 35 conventional, 36 navigated.

24. Zhu X, Qian Y, Liu A, Xu P, Guo JJ. Comparative outcomes of patient-specific instrumentation, the conventional method and navigation assistance in open-wedge high tibial osteotomy: A prospective comparative study with a two-year follow up. *Knee*. 2022;39:18-28. doi:[10.1016/j.knee.2022.08.013](https://doi.org/10.1016/j.knee.2022.08.013)

Non randomized. 144 patients included, 144 HTO : 48 conventional, 48 navigated, 48 PSI.

2) General details


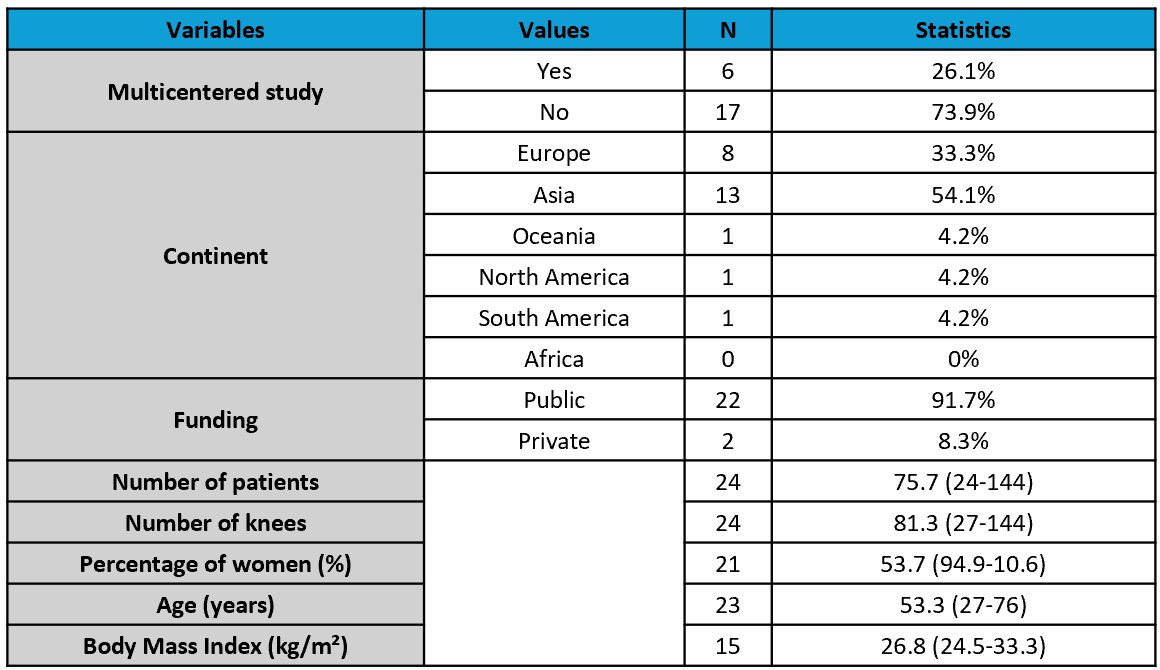


3) Surgical details


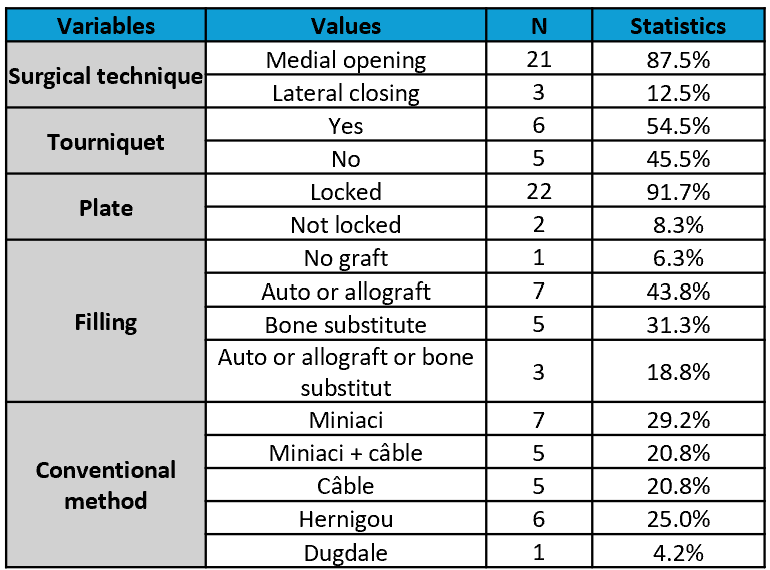

Supplement: Supplementary file 2 — Supplementary Material 2: Appendix 2: details about included studies. References, general details and surgical details [file 43019_2025_278_MOESM2_ESM.docx]
